# Complete Genome Sequences of Four Foot-and-Mouth Disease Viruses of Serotype South African Territories 1 (SAT 1), Topotype X, Isolated from Cattle in Nigeria in 2015

**DOI:** 10.1128/genomeA.01065-17

**Published:** 2017-10-19

**Authors:** Frank Vandenbussche, Elisabeth Mathijs, Hussaini G. Ularamu, David O. Ehizibolo, Andy Haegeman, David Lefebvre, David D. Lazarus, Yiltawe S. Wungak, Annebel R. De Vleeschauwer, Steven Van Borm, Kris De Clercq

**Affiliations:** a Molecular Platform, Veterinary and Agrochemical Research Centre, Ukkel, Belgium; b FMD Laboratory, Viral Research Division, National Veterinary Research Institute (NVRI), Vom, Nigeria; c Vesicular and Exotic Diseases Unit, Veterinary and Agrochemical Research Centre, Ukkel, Belgium

## Abstract

The complete genome sequences of four foot-and-mouth disease viruses of South African territories 1 (SAT 1) serotype are reported. These viruses originate from an outbreak in Nigeria in 2015 and belong to the novel SAT 1 topotype X from the west and central African virus pool.

## GENOME ANNOUNCEMENT

Foot-and-mouth disease virus (FMDV) is a positive-sense, single-stranded RNA virus of the genus *Aphthovirus*, family *Picornaviridae* that causes a highly contagious vesicular disease in cloven-hooved animals. The virus is classified into 7 immunologically distinct serotypes (O, A, C, Asia 1, South African territories 1 [SAT 1], SAT 2, and SAT 3), each containing numerous genetically and geographically distinct evolutionary lineages or topotypes (1). Recently, the novel SAT 1 topotype X was described in the west and central African FMDV virus pool (2).

Here, we report the complete genome sequences of four FMDV SAT 1 viruses that were isolated from epithelial tissue samples from cattle showing typical FMD lesions. All samples were collected from cattle herds located in Plateau State (Nigeria) during the 2015 outbreak. Total RNA was extracted from cell culture supernatant with a NucleoSpin RNA virus kit (Macherey Nagel) and treated with Baseline-ZERO DNase (Epicentre) to remove host DNA. cDNA was synthesized according to the manufacturer’s instructions using SuperScript IV reverse transcriptase (Thermo Fisher Scientific), an anchored oligo(dT) primer, and an FMDV-specific internal primer. Sequencing libraries were prepared using a Nextera XT kit (Illumina) as described by the user’s manual. The fragment length distributions of the libraries were verified on a Bioanalyzer System (Agilent Technologies), and libraries were quantified using a KAPA library quantification kit (Kapa Biosystems). Sequencing was performed on a MiSeq system using MiSeq reagent kit v3 (2 × 300 bp, Illumina).

Raw reads were trimmed using Trimmomatic (version 0.36) with a quality cutoff of 15, sliding window of 4 bp, and minimum length cutoff of 50 bp (3). *De novo* assembly of the L fragment was performed using IVA (version 1.0.8) (4) and SPAdes (version 3.9.0) (5). The S fragment and the beginning and end regions of the L fragment were verified with Sanger sequencing. The genomes were annotated using GATU, with the FMDV strain SAT1-7isrl4/62 serving as reference genome (GenBank accession number AY593844).

Complete genome sequences were obtained for all four isolates with lengths varying from 8,217 to 8,220 bp. The first 3 to 4 nucleotides of the genomes and the composition or length of the poly(C) tract could not be determined. The genomes were predicted to contain a single open reading frame (ORF) of 7,017 nt encoding a polyprotein of 2,338 amino acids.

### Accession number(s).

The nucleotide sequences for FMDV SAT1/NIG/1/15, SAT1/NIG/2/15, SAT1/NIG/3/15, and SAT1/NIG/4/15 have been deposited in GenBank under accession numbers MF678823 to MF678826.
